# Effects of an outdoor horticultural activities program on cognitive and behavioral functioning, mood, and perceived quality of life in people with dementia: a pilot study

**DOI:** 10.3389/fpsyg.2023.1182136

**Published:** 2023-06-09

**Authors:** Erika Borella, Andrea Melendugno, Chiara Meneghetti, Veronica Murroni, Elena Carbone, Giulia Goldin, Raffaele Cavalli, Andrea Basso, Francesca Pazzaglia

**Affiliations:** ^1^Department of General Psychology, University of Padova, Padova, Italy; ^2^Inter-University Research Center in Environmental Psychology (CIRPA), Rome, Italy; ^3^Casa Madre Teresa di Calcutta (O.P.S.A.), Padova, Italy; ^4^Department of Land Environment, Agriculture and Forestry, University of Padova, Padova, Italy; ^5^“Giotto” Social Cooperative, Padova, Italy

**Keywords:** horticultural therapy, psychosocial interventions, dementia, cognitive functioning, mood, behavioral functioning

## Abstract

**Introduction:**

One of various non-pharmacological treatments for people with dementia (PwD) is horticultural therapy. The aim of this double-blind, pre- and post-test, pilot study was to examine the effects of horticultural activities (HA) for PwD at a residential and daytime care facility. Whether combining HA with elements drawn from other psychosocial interventions (cognitive stimulation) would maximize any benefits was also newly examined.

**Materials and methods:**

Twenty-four PwD were involved either in HA, alone (TG1, *N* = 7) or combined with some cognitive stimulation (TG2, *N* = 8), or in indoor treatment-as-usual activities (CG, *N* = 9). Benefits were assessed in terms of general cognitive functioning (for participants with mild-to-moderate dementia), mood, behavioral and psychological symptoms, and quality of life.

**Results:**

No differences emerged between TG1 and TG2 in any outcome measure, so the two groups were combined (*N* = 15). Compared with the CG, the TG involved in HA exhibited less frequent and severe behavioral and psychological symptoms and an improved mood after the intervention. Caregivers also reported less distress in the TG after the intervention than in the CG. Considering only PwD with mild-to-moderate dementia, the TG also showed benefits in a measure of general cognitive functioning, and self-reported quality of life, compared with the CG.

**Discussion:**

Our results further confirm that engaging PwD in participatory HA in contact with natural elements can decrease their dementia symptoms and their caregivers’ distress, but also increase PwD’s quality of life. Our findings also suggest the need to consider dementia severity when assessing the benefits of horticultural therapy.

## 1. Introduction

A crucial issue associated with longevity concerns the rising incidence of neurocognitive disorders in the older adult population. Dementia has become a major cause of disability and dependence, affecting nearly 50 million people worldwide (Alzheimer’s Disease International, 2019). People with dementia (PwD) experience a gradual decline in their cognitive functioning (e.g., memory, orientation, and learning capacity), and a deterioration in their emotional, social, and behavioral control that affects their day-to-day autonomy. Given the devastating impact of the disorder on the quality of life (QoL) of PwD and their caregivers, and its cascading social and economic implications, the need to respond to such a global public health priority with effective therapeutic approaches has become urgent. While researchers are still trying to develop disease-modifying pharmacological therapies, systematic reviews (Abraha et al., 2017; McDermott et al., 2018) and guidelines (National Institute for Health and Care Excellence, 2018) currently recommend non-pharmacological approaches for PwD.

Among various psychosocial interventions (e.g., cognitive stimulation, reminiscence, and music therapy Lobbia et al., 2019), there are also programs revolving around the restorative and stimulating potential of interaction with natural elements to alleviate behavioral and psychological symptoms of dementia, and improve the QoL of PwD (Murroni et al., 2021). In particular, there is accumulating evidence of horticultural therapy, i.e., participatory horticulture-related activities, being able to positively affect psychological health outcomes not only in typically-aging older adults (Lin et al., 2022), but also in PwD (Lu et al., 2020; Zhao et al., 2022). Therapeutic horticultural activities (HA) are typically conducted in small groups. PwD can engage in simple actions (e.g., touching, smelling, and tasting edible plants) or more complex activities (e.g., picking seeds, gardening, growing, cooking, and eating their own vegetables). HA can be safe, meaningful and familiar to many PwD. They can generate valuable end products, and be a source of multisensory stimulation (colors, structures, scents, tastes, shapes, and sounds). HA exercise physical and cognitive skills (memory, orientation), they can prevent emotional outbursts, and create opportunities for a meaningful engagement and social interaction between PwD and with their carers (Jarrott et al., 2002; Ferrini, 2003).

The extant horticultural therapy-based interventions for PwD (Lu et al., 2020; Murroni et al., 2021; Zhao et al., 2022) vary considerably in the types of activity proposed, and the facilitators or staff members involved (e.g., some activities require one-on-one assistance; Jarrott et al., 2002; Gigliotti et al., 2004; Gigliotti and Jarrott, 2005; Jarrott and Gigliotti, 2010; Hewitt et al., 2013). Studies on horticultural therapy also differ in the outcomes considered and their assessment. Some used *ad-hoc* observational tools to record behaviors and engagement of PwD during or right after HA-based interventions (Jarrott et al., 2002; Gigliotti et al., 2004; Gigliotti and Jarrott, 2005; Park et al., 2008; Jarrott and Gigliotti, 2010; Hall et al., 2018). Others used various questionnaires to measure changes in specific behavioral symptoms, such as agitation (Lee and Kim, 2008; Luk et al., 2011), apathy (Yang et al., 2022), or vitality (Masuya and Ota, 2014). Not many studies included measures of general cognitive functioning (Luk et al., 2011; Hewitt et al., 2013; Masuya and Ota, 2014; Yang et al., 2022) or QoL (Hewitt et al., 2013; Yang et al., 2022), and very few included a proper treatment-as-usual control condition, making it impossible to fully capture HA-related gains (Jarrott and Gigliotti, 2010; Luk et al., 2011).

Overall, despite the promising results obtained with HA for managing dementia symptoms and supporting QoL in PwD, none of the previous studies jointly examined their impact on cognitive and behavioral symptoms, mood and QoL of PwD, and their caregivers. Nor did they more comprehensively combine HA with elements drawn from other effective psychosocial therapies, such as cognitive stimulation interventions (see below; Abraha et al., 2017; McDermott et al., 2018) to try and maximize their effects, and this is an issue worth investigating.

The aim of the present pilot study was therefore to assess the effects of HA on PwD at a residential and daytime care facility, newly examining their benefits by jointly considering the following domains typically targeted by psychosocial interventions, assessed with gold-standard tools: (i) general cognitive functioning the Montreal Cognitive Assessment (Nasreddine et al., 2005); the Alzheimer’s Disease Assessment Scale—Cognitive subscale (Rosen et al., 1984); (ii) mood (the Cornell Scale for Depression in Dementia; Alexopoulos et al., 1988); (iii) behavioral and psychological symptoms (the Neuropsychiatric Inventory; Cummings et al., 1994); and (iv) QoL (the QoL-Alzheimer’s Disease scale; Logsdon et al., 1999). Furthermore, we used a double-blind design and included a treatment-as-usual control (TAU) condition to better capture HA-related gains, issues rarely considered in previous research (Lu et al., 2020; Zhao et al., 2022).

The HA program was developed by various experts (psychologists specialized and dementia and cognitive stimulation therapies, botanists, and gardeners), drawing on the existing literature as regards the duration of the program, the selection of appropriate natural elements (plants, seeds, and bulbs). The HA were chosen for their multidimensional (sensory, perceptive, emotional, and social) stimulation potential, cost-effectiveness, seasonality, rapid germination, and growth (to obtain an end product quickly and easily), familiarity, versatility, and safety.

A further aim was to examine whether and to what extent including features and elements drawn from other effective psychosocial interventions for dementia—especially evidence-based cognitive stimulation programs like Cognitive Stimulation Therapy (CST; Carbone et al., 2021; Woods et al., 2023)—could promote the benefits afforded by HA. These features are indeed thought to make the stimulation activities more effective and facilitate the engagement of PwD in the proposed activities. Spatial–temporal orientation activities for individuals and groups, and external cues or implicit learning modalities, as used in CST, were therefore newly incorporated in our structured HA program yielded to one group of participants, to potentially maximizing its benefits.

In line with previous evidence (Lu et al., 2020; Zhao et al., 2022), we expected the HA program to be more effective than TAU in decreasing behavioral and depressive symptoms, and agitation (Zhao et al., 2022). As for the impact of HA on the other symptoms explored, we could also expect benefits in general cognitive functioning and QoL. We also investigated whether drawing on elements from other cognitive stimulation interventions could lead to greater benefits than the structured HA alone.

## 2. Materials and methods

### 2.1. Participants

The sample was recruited at a residential care home in Northern Italy. Eligibility was restricted to individuals with: (i) a diagnosis of major neurocognitive disorder (of any etiological subtype) according to the Diagnostic and Statistical Manual of Mental Disorders (DSM-5); (ii) age over 65 years; (iii) no learning disability, current physical illness or impairment, and no diagnosed comorbid psychiatric disorders or severe behavioral symptoms that might affect participation; and (iv) autonomous locomotion, or supported by mobility aids.

Twenty-four eligible individuals were identified, 16 in the mild-to-moderate and eight in the severe stages of dementia.^1^ Participants were allocated in equal proportions to three conditions: one group was involved in the structured HA program (TG1; *N* = 7; all females); one group attended the same HA program with added elements drawn from CST (TG2; *N* = 8; seven females); and one group joined typical indoor educational activities for control purposes (CG; N = 9; six females).

Table 1 shows the descriptive statistics of the sample’s demographics by group. No significant differences emerged between the groups in terms of age [total sample: H_(2)_ = 1.71, *p* = 0.45; mild-to-moderate dementia only: H_(2)_ = 3.13, *p* = 0.19] or education [total sample: H_(2)_ = 3.36, *p* = 0.18; mild-to-moderate dementia only: H_(2)_ = 4.73, *p* = 0.11].

**Table 1:** Descriptive statistics of participants’ demographic characteristics by group (the whole sample, and only participants with mild-to-moderate dementia).

		Whole sample			Participants with mild-to-moderate dementia		
		*N*	*M*	*SD*	*N*	*M*	*SD*
TG1	Age	7	79.14	5.64	4	79.00	4.96
	Education (years)	7	7.57	5.09	4	9.25	6.23
TG2	Age	8	81.25	5.17	7	81.29	5.58
	Education (years)	8	4.38	2.32	7	4.29	2.49
CG	Age	9	82.67	6.91	5	86.00	3.00
	Education (years)	9	7.88	3.64	5	8.40	2.88

TG1, structured horticultural activities; TG2, structured horticultural activities + cognitive stimulation; and CG, treatment as usual.

### 2.2. Materials

#### 2.2.1. General cognitive functioning

The *Montreal Cognitive Assessment* (MoCA; Nasreddine et al., 2005) comprises items testing visuospatial abilities, executive functions, language, delayed memory recall, attention, and temporal and spatial orientation. The dependent variable was the sum of the scores (max. 30), corrected for age and education.

The *Alzheimer’s Disease Assessment Scale—Cognitive subscale* (ADAS-Cog; Rosen et al., 1984) consists of 11 tasks that assess orientation, memory, language, praxis, attention, and other cognitive abilities. The dependent variable was the sum of the scores (max. 70), with higher scores indicating a more impaired cognitive functioning.

#### 2.2.2. Mood

The *Cornell Scale for Depression in Dementia* (Cornell; Alexopoulos et al., 1988) consists of 19 items that assess signs and symptoms of major depression in individuals with dementia. Each item is rated for severity on a scale from 0 (absent) to 2 (severe). The dependent variable was the sum of the scores for the 19 items, with higher scores indicating more severe depressive symptoms.

#### 2.2.3. Behavior

The *Neuropsychiatric Inventory* (NPI; Cummings et al., 1994) assesses 12 behavioral and psychological disturbances in dementia patients (delirium, hallucinations, agitation, dysphoria, anxiety, euphoria, apathy, disinhibition, irritability, motor disturbances, sleep disturbances, and food issues). For each disturbance, it is also possible to assess the emotional and psychological distress experienced by the caregiver. The dependent variables were: (i) the sum of the frequency × severity scores on each symptom; and (ii) the sum of the caregiver’s distress scores on each symptom. Higher scores correspond to more frequent and more severe disturbances and more severe caregiver distress.

#### 2.2.4. Quality of life

The *Quality of Life—Alzheimer’s Disease scale* (QoL-AD; Logsdon et al., 1999) is a 13-item questionnaire assessing subjective components (e.g., perceived QoL and psychological well-being), and objective components (e.g., behavioral competence and environment) of QoL, rated by caregivers and participants on a four-point scale from 1 (poor) to 4 (excellent). The dependent variables were the sums of all the items separately rated by caregivers and participants, where higher scores indicate a better QoL.

### 2.3. Procedure

**Figure 1:**
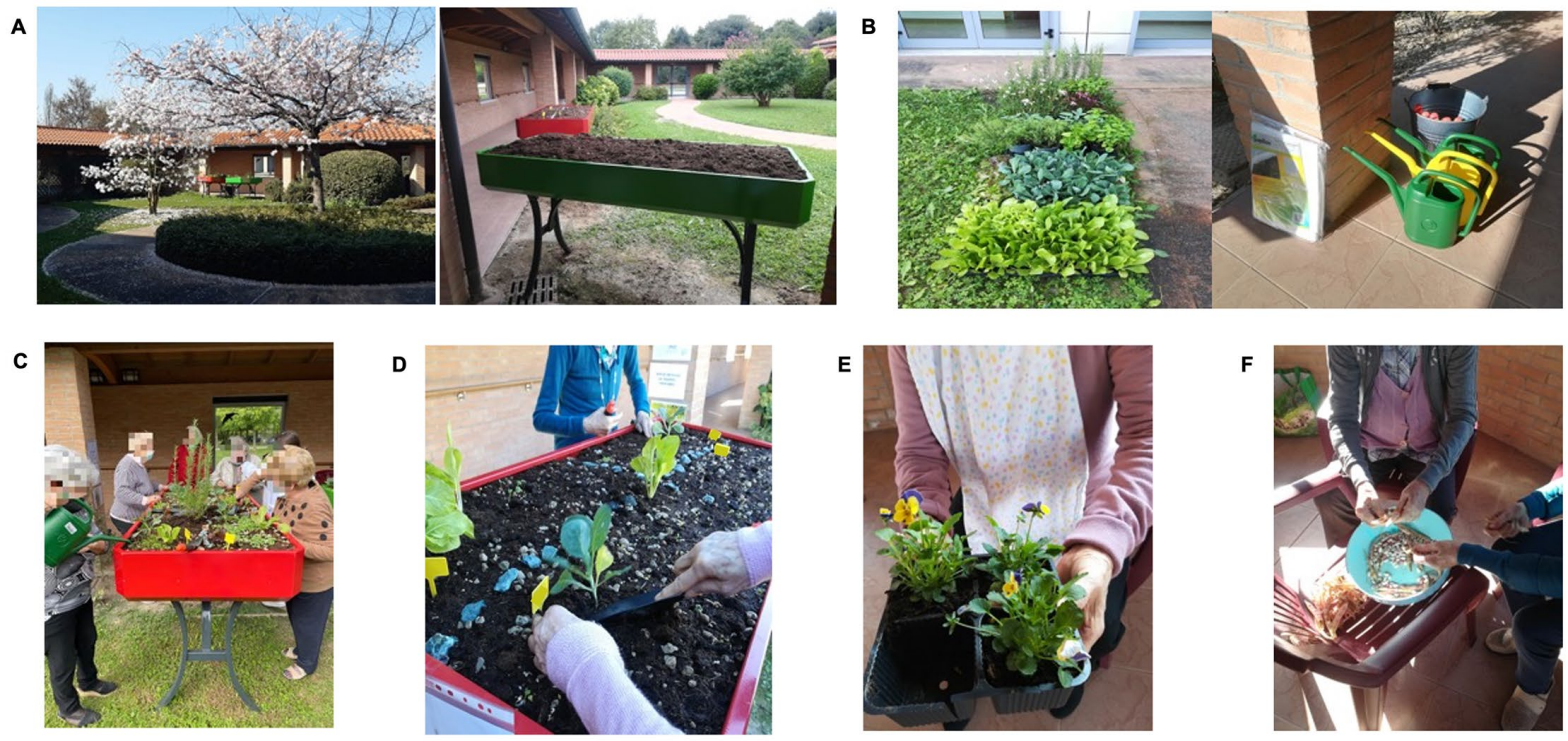
Pictures of the garden area with the raised garden beds **(A)** and materials **(B)** used for horticultural activities, and example of horticultural activities: planting and watering aromatic plants **(C)**; transferring horticultural species **(D)**; exploring colorful flowers **(E)**; and shelling beans **(F)**.

All participants attended 14 sessions. The first and last were pre- and post-test individual sessions lasting an hour each to complete a battery of tests and questionnaires (see Table 2). Caregivers (staff members, or informal caregivers for PwD attending the facility during the day) were also involved in completing some of the questionnaires (NPI, Cornell, and QoL-AD). The other 12 were group sessions lasting about 40 min each, delivered twice a week for 6 weeks in small groups (3–5 people). During these sessions, the TGs took part in the structured HA programs in a therapeutic garden (see Meneghetti et al., 2023) area just outside the facility. This area was readily accessible and visible, also from inside the care home. It was on level ground partially shaded by a covered walkway. There were also chairs for those who wanted to carry out the activities sitting down. Raised garden beds were located in a partly-paved area, and PwD who used aids such as walkers and wheelchairs were given a place with a leveled paved floor to avoid falls (see Figure 1).

**Table 2:** Content and structure of the sessions, themes, and main horticultural activities for the two programs.

Pre-test session	ADAS-Cog; MoCA; QoL-AD (for PwD); NPI, CSDD; QoL-AD (for caregiver)	
	Structured horticultural activities	Structured horticultural activities + cognitive stimulation
Fixed structure of each session	·Introduction with a general welcome and a starting ritual that involves removing the protective sheet from the raised garden beds, checking for changes, cleaning and watering (5 min).	·Introduction with a personalized welcome: *discussion on choosing a group name (only at the first session; the group name is then used for the following sessions), space–time orientation activities, the previous activity is resumed and a new one is presented*; contextualized ritual (removing the protective sheet from the raised beds; 5 min).
	·The facilitator presents the activity for the session underway, and gives instructions on how to complete it (30 min).	·Main horticultural activities: the facilitator presents the activity for the session, *explores preferences, local traditions, and customs related to the plant involved,* and gives instructions on how to complete the activity (30 min).
	·Ending ritual that involves covering the garden beds with the protective sheet and reminding participants about the next session (5 min).	·Conclusion: *using laminated images of the various activities proposed during the session, the facilitator sums up, step by step, what has been done, asks for session feedback, thanks everyone for participating*; the ending ritual then involves covering the garden beds with the protective sheet and reminding participants about the next session (5 min).
Session themes and horticultural activities (common to both programs):		
Session 1—Arranging the raised garden beds	Choosing workstation; pouring in potting soil; arranging soil with trowels; moistening topsoil with watering cans; and decorating and surrounding garden with different-colored stones and signs.	
Session 2—Seeds	Seeing seeds; handling seeds; seeing images of end product; choosing seeds; planting seeds; watering the garden; and covering with non-woven protective sheet.	
Session 3—Exploring horticultural species for transfer	Seeing, touching, and smelling plants.	
Session 4—Transferring plants	Choosing a plant; making a hole in the soil; taking the plant out of the pot; putting the plant in the hole and surrounding it with soil; and watering the plant.	
Session 5—Exploring aromatic plants	Seeing, touching, and smelling aromatic plants.	
Session 6—Transferring aromatic plants	Choosing a plant; making a hole in the soil; taking the plant out of the pot; putting the plant in the hole and surrounding it with soil; and watering the plant.	
Session 7—Exploring colorful flowers	Seeing, touching, and smelling colorful flowers.	
Session 8—Transferring colorful flowers	Choosing a plant; making a hole in the soil; taking the plant out of the pot; putting the plant in the hole and surrounding it with soil; and watering the plant.	
Session 9—Shelling beans	Seeing and touching (observing and shelling) beans; delivering shelled beans to the kitchen; and eating beans on the weekly menu.	
Session 10—Shelling other legumes	Seeing, touching other legumes, manual activity; delivering legumes to the kitchen; and eating the legumes on the weekly menu.	
Session 11—Harvesting aromatic plants	Collecting and drying twigs and leaves of aromatic plants; delivering part of the harvest to the kitchen.	
Session 12—Harvesting plants previously planted or grown from seed	Arranging flowers; collecting plants that had previously been planted or grown from seed; and delivering part of the harvest to the kitchen.	
Post-test session	ADAS-Cog; MoCA; QoL-AD (for PwD); NPI, CSDD; and QoL-AD (for caregiver).	

ADAS-Cog, Alzheimer’s disease assessment scale-Cognitive subscale; MoCA, montreal cognitive assessment; NPI, neuropsychiatric inventory; QoL-AD, quality of life—Alzheimer’s disease scale; CSDD, Cornell scale for depression in dementia. In italics, the activities/approach that differ between the two programs.

Each session revolved around a specific horticultural theme and related main activities (see Table 2 for the structure of the program and the specific activities). Different types of plants (edible, aromatic, colorful flowering plants, and legumes) were chosen to provide a variety of sensory (tactile, olfactory, and visual) stimuli. The HA ranged from simple actions (e.g., touching, smelling, and examining plants) to more complex ones (e.g., planting, watering), providing variable degrees of social and cognitive stimulation in the different session (see Table 2). The end products of some sessions (9–12) were then delivered to the residential care home kitchen for cooking (see Figure 1).

Each group’s sessions were based on the same format, which differed between TG1 and TG2 (see Table 2). The HA sessions with features drawn from other cognitive stimulation interventions started with personal and spatial–temporal orientation activities, and a summary of the activities involved in the previous session. They ended with the facilitator summing up the main activities of the ongoing session with the aid of laminated images depicting the various activities proposed, and then completing a final ritual and reminding participants about the next session (see Table 2). The HA were delivered to the two TGs by the same facilitator, a trained psychologist with experience of dementia care and group facilitation, and of the person-centered approach characteristic of psychosocial, cognitive stimulation interventions. The control group was involved in the care home’s usual group-based indoor educational activities (e.g., gentle gymnastics, music therapy, watching historical documentaries and films, and Reality Orientation Therapy) for the same number of sessions, and with the same amount of time and interaction with a trained and expert facilitator.

## 3. Results

All analyses were run using non-parametric statistics and considering the sample as a whole, and then separately analyzing the individuals with mild-to-moderate dementia.

First of all, at pre-test, we checked for any baseline differences between the three groups. The results of the Kruskal-Wallis test revealed no differences between them in any of the measures of interest, neither in the sample as a whole, nor for the individuals with mild-to-moderate dementia (see Supplementary Tables S1, S2), who were also tested on measures of general cognitive functioning and perceived QoL.

Then, differences between the two treatment groups (TG1 and TG2) in terms of cognitive, behavioral, and psychological symptoms, mood, and QoL were examined, computing gain scores (post-test—pre-test) for each measure of interest. The Mann–Whitney test showed no significant differences between the gain scores in TG1 and TG2 for any of the outcome measures considered (except for a lesser frequency and severity of agitation in TG2 when only participants with mild-to-moderate dementia were considered; see Supplementary Table S3).

Based on these results, we merged the two TG groups together (to obtain a larger sample size in the subsequent analyses). Supplementary Tables S4, S5 show descriptive statistics of the measures of interest by group, for the whole sample and for people with mild-to-moderate dementia, and the results show that there were no differences between the two TG groups.

### 3.1. Differences in gain scores between TG and CG

Differences between the TG and CG groups in terms of cognitive, behavioral and psychological symptoms, mood, and QoL were examined, computing the gain scores (post-test score—pre-test score) for each measure of interest (see Table 3).

**Table 3:** Descriptive statistics for the gain scores (post-test score—pre-test score) for each measure of interest by group (TG: participants involved in horticultural activities; CG: treatment-as-usual), results from the Mann–Whitney test for the differences between the treatment and control groups in gain scores for each measure of interest and Cohen’s *d*, for the total sample and participants with mild-to-moderate dementia only.

	Whole sample (*N* = 24)								Participants with mild-to-moderate dementia (*N* = 16)							
	TG (*N* = 15)		CG (*N* = 9)		Gain scores differences		Cohen’s *d*		TG (*N* = 11)		CG (*N* = 5))		Gain cores differences		Cohen’s *d*	
	*M*	*SD*	*M*	*SD*	*Z*	*p*	TG	CG	*M*	*SD*	*M*	*SD*	*Z*	*p*	TG	CG
ADAS-Cog									−3.51	7.79	−1.04	8.41	−0.31	0.76	−0.45	−0.09
MoCA									1.27	2.41	−2.40	2.61	−2.28	0.02	0.37	−0.60
*NPI—Delirium*	−1.40	3.23	0.78	2.11	−1.65	0.10	−0.49	0.47	−1.91	3.67	1.00	2.83	−1.27	0.20	−0.59	0.51
*NPI—Hallucinations*	−0.13	1.92	2.00	4.24	−1.38	0.17	−0.07	0.86	−0.18	2.27	3.60	5.37	−1.64	0.10	−0.08	0.63
*NPI—Agitation*	−1.13	4.02	3.33	4.72	−1.87	0.06	−0.41	0.63	−0.73	4.13	2.80	3.90	−1.04	0.30	−0.26	0.77
*NPI—Dysphoria*	−0.20	3.69	1.56	4.07	−1.07	0.28	−0.08	0.68	−0.82	3.40	2.80	5.36	−1.24	0.21	−0.36	0.51
*NPI—Anxiety*	−1.33	3.60	0.22	2.68	−0.88	0.38	−0.53	−0.20	−1.82	3.97	−0.40	3.21	−0.59	0.55	−0.64	0.13
*NPI—Euphoria*	0.20	1.08	0.78	1.20	−1.64	0.10	0.26	0.57	0.27	1.27	0.40	0.89	−0.75	0.46	0.29	0.87
*NPI—Apathy*	−0.93	3.97	0.00	5.98	−0.50	0.62	−0.23	−0.68	−0.36	3.41	−3.00	4.69	−0.89	0.37	−0.09	0.00
*NPI—Disinhibition*	−0.47	0.92	−0.22	3.27	−0.29	0.77	−0.39	−0.07	−0.73	0.91	−0.20	4.27	−0.43	0.67	−0.77	−0.10
*NPI—Irritability*	0.07	2.49	1.33	4.80	−1.26	0.21	0.03	0.92	0.45	2.77	3.60	4.98	−1.75	0.08	0.23	0.40
*NPI—Motion*	−2.07	3.88	−1.11	3.33	−0.64	0.52	−0.44	−0.08	−1.91	4.28	−0.40	2.97	−0.47	0.64	−0.43	−0.21
*NPI—Sleep*	0.47	1.19	3.78	5.14	−1.51	0.13	0.35	0.85	0.27	0.79	4.40	4.98	−1.81	0.07	0.31	0.83
*NPI—Food*	−0.40	3.36	0.22	3.03	−0.28	0.78	−0.18	0.24	−0.09	3.78	0.80	4.09	−0.40	0.69	−0.04	0.08
NPI-symptoms	−7.33	15.26	12.67	18.47	−2.33	0.02	−0.59	0.70	−7.55	17.97	15.40	23.58	−2.45	0.01	−0.54	0.78
NPI-distress	−2.80	7.54	4.67	6.91	−2.49	0.01	−0.39	0.93	−3.45	8.70	7.80	7.92	−1.53	0.13	−0.43	0.67
QoL-AD (cgv)	0.47	5.50	−1.33	3.97	−0.81	0.42	0.07	−0.32	0.45	5.92	−2.00	4.64	−0.86	0.39	0.06	−0.28
QoL-AD (PwD)									1.36	3.11	−4.60	5.41	−2.05	0.04	0.33	−0.88
CSDD	−2.53	3.62	1.00	2.35	−2.53	0.01	−0.70	0.27	−2.18	3.82	1.20	2.95	−1.60	0.11	−0.56	0.23

TG, participants involved in structured horticultural activities; CG, treatment-as-usual; ADAS-Cog, Alzheimer’s disease assessment scale-cognitive subscale; MoCA, montreal cognitive assessment; NPI, neuropsychiatric inventory; QoL-AD, quality of life—Alzheimer’s disease scale; and CSDD: Cornell scale for depression in dementia.

When the whole sample was considered, the results of the Mann–Whitney test showed that the TG exhibited less frequent and severe behavioral and neuropsychiatric symptoms (NPI total score), and fewer depressive symptoms (CSDD scores) than the CG (see Table 3). The TG’s caregivers also reported lower levels of distress related to the behavioral and psychological symptoms of the PwD in their care (as measured by the NPI) than the CG’s caregivers (see Table 3).

When participants with mild-to-moderate dementia were considered separately, our findings confirmed the TG’s less frequent and severe behavioral and neuropsychiatric symptoms, compared with the CG. The results also showed that, by comparison with the CG, the TG gained significantly from pre-test to post-test in one of the general cognitive functioning measures (the MoCA) and in terms of self-reported QoL (QoL-AD scores; see Table 3).

### 3.2. Effect sizes

To gain a better understanding of the dimensions of the benefits obtained with the HA, effect sizes (Cohen’s *d*) were computed separately for the TG and CG (see Table 3).

For the TG, medium effect sizes emerged both for the frequency and severity of behavioral and psychological symptoms (*d* = −0.59), and for caregiver distress (*d* = −0.39) total scores on the NPI, as well as for the frequency and severity of specific behavioral and psychological symptoms, i.e., delirium (*d* = −0.49); agitation (*d* = −0.41); anxiety (*d* = −0.53); disinhibition (*d* = −0.39); and motor disturbances (*d* = −0.44). The effect size was large for the measure of depressive symptoms (CSDD; *d* = −0.70). The pattern of results was broadly the same when participants with mild-to-moderate dementia were considered separately, with medium effect sizes for NPI total scores (frequency and severity of behavioral and psychological symptoms*: d* = −0.54; caregiver distress: *d* = −0.43) and depressive symptoms (CSDD; *d* = −0.56). Medium effect sizes also emerged for the two general cognitive functioning measures (ADAS-Cog: *d* = −0.45; MoCA, *d* = 0.37), and for self-reported QoL, as measured by the QoL-AD (*d* = 0.33; see Table 3).

Whether the whole sample was considered, or only the participants with mild-to-moderate dementia, the effect sizes for the CG revealed a worsening picture, particularly in the frequency and severity of behavioral and psychological symptoms, distress, and QoL, as reported by caregivers (medium to large effect sizes; see Table 3). For participants with mild-to-moderate dementia, there were also medium effect sizes for the MoCA, indicating a deterioration in general cognitive functioning and perceived QoL. There was no change for the mood measure (small effect size; see Table 3).

## 4. Discussion

This pilot study examined the beneficial effects of involving PwD at a residential and daytime care facility in outdoor participatory HA. A new structured 2-month HA program was developed and its effects on cognitive, behavioral and psychological functioning in PwD, and on the QoL of both the PwD and their caregivers (in terms of distress) were examined. Besides including a TAU control condition, rarely used in this type of intervention, we also made a first attempt to examine whether including elements drawn from other cognitive stimulation programs (Carbone et al., 2021; 2022) for dementia could maximize its benefits.

Our findings did not reveal any clear differences between the groups of PwD who engaged in HA alone (TG1) or in HA combined with elements drawn from other cognitive stimulation programs (TG2). Although in contrast with our expectations, this result may be because the characteristics of the two programs differed only slightly. Although participants in the TG2 were given additional cognitive stimulation (e.g., personal/group and spatial–temporal orientation activities, external cues, or implicit learning modalities) to promote their engagement in the proposed activities, these features were mainly used at the beginning and at the very end of the sessions. The structure of the sessions was broadly the same for the TG1 and TG2, and the HA forming the main part of each session overlapped. The same trained facilitator also managed the sessions for both groups, and this is another aspect that might have prevented us from capturing any differences between the two programs. Future studies should, thus further explore the potential additive effects of incorporating elements drawn from other cognitive stimulation interventions for dementia in participatory HA, by involving PwD at different facilities, and different facilitators, for example.

Interesting results emerged, however, when the PwD involved in HA were compared with the TAU controls. Participants in the TG exhibited a reduction in the frequency and severity of their behavioral, psychological and depressive symptoms from pre-test to post-test, when compared with the CG. Lower levels of distress were also reported by their caregivers, compared with the CG’s caregivers. The same pattern of results emerged when only participants with mild-to-moderate dementia were considered (apart from caregivers’ distress ratings).

When only PwD with mild-to-moderate dementia were considered, there was also an improvement in one of the general cognitive functioning measures (the MoCA), corroborating the “cognitively stimulating” impact of HA (Lu et al., 2020; Zhao et al., 2022). No such benefit emerged for the other general cognitive functioning measure considered here (the ADAS-Cog), however. The reason for such inconsistent findings might lie in the characteristics of the two measures: both cover cognitive domains, but the ADAS-Cog taxes some (i.e., comprehension and memory) more specifically, while the MoCA does not. The former may therefore be less sensitive to the multidimensional stimulation of nature-based HA for PwD. Future studies should attempt to include a more comprehensive battery of cognitive tasks to gain a more detailed understanding of which cognitive domains and cognitive mechanisms might benefit most from exposure to natural elements and HA—an issue rarely examined in older age, and never in PwD (Ricciardi et al., 2022).

As expected, our PwD with mild-to-moderate dementia also gained from HA in terms of perceived QoL, confirming previous evidence (Lu et al., 2020; Zhao et al., 2022).

The effect sizes corroborated our findings, and—although descriptively—indicated more nuanced benefits for the TG, especially in terms of a decreased frequency and severity of behavioral and psychological symptoms as delirium, agitation, anxiety, disinhibition, and motor disturbances, both for the whole sample and for participants with mild-to-moderate dementia. They revealed a worsening particularly in the frequency and severity of behavioral and psychological symptoms, instead, for the CG.

Interestingly, the effect sizes indicated an increasing frequency and severity of sleep disorders from pre- to post-test in the TG, and even more so in the CG. Sleep disturbances are among the first symptoms of dementia. They have various causes, some of them biologically based (e.g., disrupted circadian rhythms or damaged neuronal pathways; Vitiello and Borson, 2001), which might make them harder than other symptoms to counteract with non-pharmacological interventions. The HA program discussed here may also have been too short to induce any change in such a symptom. Further studies are needed to clarify the effects of exposure to natural elements and HA on sleep patterns in PwD (Lee and Kim, 2008), and on their other behavioral/psychological symptoms.

Taken together, these results, in line with previous reports and our expectations, confirm that engaging PwD, regardless of dementia severity, in participatory HA alleviates their behavioral disturbances and supports their mood (Lu et al., 2020; Murroni et al., 2021; Zhao et al., 2022). Such a beneficial effect of HA might lie in the promotion of contact with natural elements. It is well known that humans are instinctively attracted to the natural world for its esthetic and fascinating features (biophilia approach; Wilson, 1984). Contact with natural elements has a restorative potential, facilitating the retrieval of resources that promote positive affective states and reduce stress (Murroni et al., 2021). HA also represent activities that PwD can manage irrespective of any prior expertise and life experiences, and that are meaningful, enable them to monitor their progress (as their plants grow) and obtain a tangible end product (Jarrott et al., 2002; Gigliotti and Jarrott, 2005). These features seem to prompt self-efficacy, a sense of ownership (participants each took care of their own plants in the garden beds), and a sense of purpose and mastery of the environment (Jarrott et al., 2002; Gigliotti and Jarrott, 2005). Although these are just speculations, all these aspects of the proposed HA may have contributed to the behavioral and psychological improvements detected in our sample of PwD.

Notwithstanding these positive results, our study has several limitations. First, our sample size was small—as in most previous studies on this topic (Lu et al., 2020)—, and recruited in a single residential care setting. It was also mostly composed by women, which however reflects the national trends (to date three out of four older adults—aged 75 years and more—living in residential care facilities in Italy are women; National Institute of Statistics, 2022) and is in line with previous studies on HA for PwD (Murroni et al., 2021). Since there is initial evidence of gender differences in terms of risk factors, clinical manifestations (e.g., slower rate of cognitive decline in women than men, higher prevalence of affective and psychotic symptoms in females and of apathy and agitation among males), and treatment response in PwD (e.g., Ferretti et al., 2020; Mielke et al., 2022), future studies should further understand whether gender differences might influence the benefits of non-pharmacological interventions of PwD, as those based on HA.

Involving people with severe dementia (i.e., unable to communicate) resulted in a lack of data for some of the outcome measures considered (cognitive measures and perceived QoL). These shortcomings are somewhat countered by our inclusion of a TAU control condition, and our use of gold-standard measures to identify any benefits of HA in different domains of functioning. As none of our participants dropped out, not even among those with severe dementia, our pilot study provides initial evidence of HA showing promise as a therapeutic approach for severely-impaired PwD too, whose symptoms and lack of communication skills often make them ineligible for psychosocial treatments (e.g., Boote et al., 2006). Nonetheless, future studies should strive to involve a larger sample of PwD to better understand whether individual differences in their profiles (e.g., stage of dementia) as well as other individual characteristics might influence the benefits afforded by HA (Carbone et al., 2022).

Moreover, although there is evidence that participatory HA—as the ones used here—seems more effective in supporting PwD’s cognitive and psychological functioning than those based on nature exposure (e.g., garden viewing, garden touring; Zhao et al., 2022), it would be interesting to compare different programs based either on HA or on nature exposure to disentangle the potential additional benefits provided by a more active engagement with nature through HA for PwD.

To better capture any benefits of HA, particularly when PwD with communication difficulties are concerned, the inclusion of observational tools or devices to detect psychological and behavioral symptoms (e.g., Tobón Vallejo and El Saddik, 2020; Ardelean and Redolat, 2023) should be considered. Follow-up assessments, which are rarely envisaged after psychosocial treatments for PwD (Hewitt et al., 2013; Masuya and Ota, 2014; Carbone et al., 2021), also warrant attention.

## 5. Conclusion

In conclusion, the results of this pilot study confirm the feasibility of our HA program, which proved easy to implement at a long-term care facility as well as appropriate for PwD at different dementia stages. The HA program showed to alleviate mood and behavioral and psychological symptoms -with beneficial effects also for carers, regardless dementia severity. They thus suggest that HA can be also proposed to people with severe dementia, who seem to benefit from it as much as those in earlier stages of the disorder. Horticultural therapy can be considered as another promising, cost-effective psychosocial approach for supporting the cognitive, mood and behavioral functioning as well as QoL of PwD and their carers.

## Data availability statement

The raw data supporting the conclusions of this article will be made available by the authors, without undue reservation.

## Ethics statement

The study was approved by the Ethical Committee of University of Padova (Univoc number: B0ED79C242237AB75E1452E090F3880A; number of protocol: 4275). All participants were informed about the purposes of the study and gave their written informed consent in accordance with the Declaration of Helsinki (World Medical Association, 2013). The patients/participants provided their written informed consent to participate in this study.

## Author contributions

EB, CM, VM, AB, AM, RC, and FP contributed to the design and implementation of the research. EB, EC, CM, and FP contributed to the analysis of the results and writing of the manuscript. GG and VM contributed to data collection. All authors contributed to the article and approved the submitted version.

## Funding

This research received specific grants from University Padova Project, Uni-Impresa (2019): “VERde e BENessere nell’Alzheimer (VER.BEN.A). Verso un modello di giardino terapeutico centrato sull’interazione luogo-persona” [Green and wellbeing in Alzheimer (Ver.Ben.a). Toward a model of therapeutic garden centered on the place-person interaction].

## Conflict of interest

The authors declare that the research was conducted in the absence of any commercial or financial relationships that could be construed as a potential conflict of interest.

## Publisher’s note

All claims expressed in this article are solely those of the authors and do not necessarily represent those of their affiliated organizations, or those of the publisher, the editors and the reviewers. Any product that may be evaluated in this article, or claim that may be made by its manufacturer, is not guaranteed or endorsed by the publisher.

## Footnotes

^1^Participants with severe dementia could no longer communicate or complete the general cognitive functioning measures and QoL questionnaire, so our results are reported for the whole sample, and separately for participants with mild-to-moderate dementia.
