# Supplementary material for: Effects of an outdoor horticultural activities program on cognitive and behavioral functioning, mood, and perceived quality of life in people with dementia: a pilot study
Source: Front Psychol. 2023 Jun 9;14:1182136. doi: 10.3389/fpsyg.2023.1182136 (PMC10390226; doi:10.3389/fpsyg.2023.1182136)
Supplement: Supplementary file 1 [file Table_1.DOCX]

**Effects of an outdoor horticultural activities program on cognitive and behavioral functioning, mood, and perceived quality of life in people with dementia: A pilot study**

Supplemental materials

Table S1. Descriptive statistics for the behavioral symptoms, quality of life and depression measures by assessment session (pre-test, post-test) and group (TG1: structured horticultural activities; TG2: structured horticultural activities + cognitive stimulation; CG: treatment-as-usual), and results from the Kruskal-Wallis test for the differences between groups at pre-test.

|  | Pre-test | | | | | | Pre-test differences | | Post-test | | | | | |
| --- | --- | --- | --- | --- | --- | --- | --- | --- | --- | --- | --- | --- | --- | --- |
|  | TG1 | | TG2 | | CG | |  |  | TG1 | | TG2 | | CG | |
|  | M | SD | M | SD | M | SD | H_(2)_ | p | M | SD | M | SD | M | SD |
| *NPI - Delirium* | 1.71 | 4.53 | 2.00 | 2.87 | 0.11 | 0.33 | 3.71 | 0.16 | 0.00 | 0.00 | 0.88 | 2.10 | 0.89 | 2.02 |
| *NPI - Hallucinations* | 0.00 | 0.00 | 1.25 | 2.37 | 0.00 | 0.00 | 4.17 | 0.12 | 0.00 | 0.00 | 1.00 | 2.82 | 2.00 | 4.24 |
| *NPI - Agitation* | 2.43 | 3.35 | 3.25 | 3.41 | 1.89 | 2.36 | 0.71 | 0.70 | 2.57 | 2.29 | 1.00 | 1.19 | 5.22 | 5.28 |
| *NPI - Disphoria* | 0.71 | 1.25 | 1.63 | 2.82 | 0.22 | 0.44 | 1.10 | 0.58 | 2.00 | 3.46 | 0.13 | 0.35 | 1.78 | 4.05 |
| *NPI - Anxiety* | 2.86 | 4.25 | 1.00 | 2.07 | 0.89 | 1.53 | 1.46 | 0.48 | 0.71 | 1.25 | 0.38 | 0.74 | 1.11 | 1.76 |
| *NPI - Euphoria* | 0.00 | 0.00 | 0.13 | 0.35 | 0.00 | 0.00 | 2.00 | 0.37 | 0.57 | 1.51 | 0.00 | 0.00 | 0.78 | 1.20 |
| *NPI - Apathy* | 4.00 | 4.00 | 2.38 | 4.40 | 2.56 | 4.39 | 0.76 | 0.69 | 1.86 | 1.86 | 2.50 | 4.75 | 2.56 | 3.97 |
| *NPI - Dishinibition* | 1.14 | 1.46 | 0.63 | 0.91 | 1.22 | 2.22 | 0.26 | 0.88 | 0.86 | 1.57 | 0.00 | 0.00 | 1.00 | 2.00 |
| *NPI - Irritability* | 1.14 | 2.26 | 1.25 | 1.38 | 1.11 | 2.02 | 0.62 | 0.74 | 2.14 | 2.96 | 0.50 | 0.92 | 2.44 | 3.97 |
| *NPI - Motion* | 8.43 | 5.02 | 1.38 | 2.32 | 4.44 | 5.81 | 5.64 | 0.06 | 4.86 | 4.45 | 0.63 | 1.40 | 3.33 | 4.24 |
| *NPI - Sleep* | 0.29 | 0.75 | 0.38 | 0.74 | 0.67 | 2.00 | 0.39 | 0.83 | 0.86 | 2.26 | 0.75 | 1.16 | 4.44 | 5.81 |
| *NPI - Food* | 2.29 | 2.43 | 0.50 | 1.41 | 1.78 | 2.27 | 3.06 | 0.22 | 0.29 | 0.48 | 1.50 | 2.82 | 2.00 | 3.16 |
| NPI- symptoms total score | 25.00 | 13.064 | 15.75 | 16.77 | 14.89 | 11.95 | 5.25 | 0.07 | 16.71 | 7.43 | 9.25 | 5.75 | 27.56 | 18.35 |
| NPI-distress total score | 5.43 | 6.42 | 7.50 | 9.91 | 4.89 | 3.72 | 0.17 | 0.92 | 4.86 | 5.04 | 2.75 | 5.87 | 9.56 | 8.58 |
| QoL-AD (caregivers) | 29.29 | 4.78 | 29.38 | 6.39 | 26.00 | 4.63 | 2.27 | 0.32 | 28.00 | 4.96 | 31.38 | 8.87 | 24.67 | 4.44 |
| CSDD | 6.57 | 3.20 | 6.75 | 5.75 | 5.22 | 3.93 | 0.80 | 0.67 | 4.14 | 1.34 | 4.13 | 2.47 | 6.22 | 4.43 |

Notes. NPI: Neuropsychiatric Inventory (scores on each symptom refers to mean frequency x severity); QoL-AD: Quality of Life - Alzheimer's Disease scale; CSDD: Cornell scale for depression in dementia.

Table S2. Descriptive statistics for each cognitive, behavioral symptoms, mood and quality of life measures of interest for participants with mild-to-moderate dementia only by group (TG1: structured horticultural activities; TG2: structured horticultural activities + cognitive stimulation; CG: treatment-as-usual), and results from the Kruskal-Wallis test for the differences between groups at pre-test.

|  | Pre-test | | | | | | Pre-test  differences | | Post-test | | | | | |
| --- | --- | --- | --- | --- | --- | --- | --- | --- | --- | --- | --- | --- | --- | --- |
|  | TG1 | | TG2 | | CG | |  |  | TG1 | | TG2 | | CG | |
|  | M | SD | M | SD | M | SD | H_(2)_ | p | M | SD | M | SD | M | SD |
| ADAS-Cog | 50.72 | 7.13 | 50.40 | 7.92 | 44.66 | 10.96 | 0.99 | 0.61 | 46.45 | 6.80 | 47.40 | 8.66 | 43.62 | 10.72 |
| MoCA | 14.06 | 1.87 | 14.22 | 3.52 | 16.36 | 2.82 | 1.89 | 0.39 | 15.06 | 4.42 | 15.65 | 3.57 | 13.96 | 4.26 |
| *NPI - Delirium* | 3.00 | 6.00 | 2.29 | 2.98 | 0.20 | 0.45 | 1.90 | 0.39 | 0.00 | 0.00 | 1.00 | 2.24 | 1.20 | 2.68 |
| *NPI - Hallucinations* | 0.00 | 0.00 | 1.43 | 2.51 | 0.00 | 0.00 | 2.74 | 0.25 | 0.00 | 0.00 | 1.14 | 3.02 | 3.60 | 5.37 |
| *NPI - Agitation* | 0.75 | 1.50 | 3.43 | 3.64 | 0.80 | 1.79 | 2.71 | 0.26 | 3.50 | 2.08 | 0.71 | 0.95 | 3.60 | 5.37 |
| *NPI - Disphoria* | 1.25 | 1.50 | 1.57 | 3.05 | 0.40 | 0.55 | 0.42 | 0.81 | 1.50 | 3.00 | 0.14 | 0.38 | 3.20 | 5.22 |
| *NPI - Anxiety* | 3.75 | 5.68 | 1.14 | 2.19 | 1.60 | 1.82 | 0.55 | 0.76 | 0.00 | 0.00 | 0.43 | 0.79 | 1.20 | 1.79 |
| *NPI - Euphoria* | 0.00 | 0.00 | 0.14 | 0.38 | 0.00 | 0.00 | 1.29 | 0.53 | 1.00 | 2.00 | 0.00 | 0.00 | 0.40 | 0.89 |
| *NPI - Apathy* | 3.00 | 3.83 | 2.71 | 4.64 | 4.60 | 5.18 | 1.20 | 0.55 | 1.75 | 2.06 | 2.86 | 5.01 | 1.60 | 2.19 |
| *NPI - Dishinibition* | 1.25 | 1.50 | 0.71 | 0.95 | 1.40 | 2.61 | 0.30 | 0.86 | 0.50 | 1.00 | 0.00 | 0.00 | 1.20 | 2.68 |
| *NPI - Irritability* | 0.50 | 1.00 | 1.14 | 1.46 | 0.40 | 0.89 | 1.23 | 0.54 | 2.75 | 3.59 | 0.57 | 0.98 | 4.00 | 4.90 |
| *NPI - Motion* | 8.75 | 4.27 | 1.57 | 2.44 | 3.20 | 5.22 | 5.34 | 0.07 | 5.00 | 5.29 | 0.71 | 1.50 | 2.80 | 3.35 |
| *NPI - Sleep* | 0.00 | 0.00 | 0.43 | 0.79 | 1.20 | 2.68 | 1.17 | 0.56 | 0.00 | 0.00 | 0.86 | 1.21 | 5.60 | 6.07 |
| *NPI - Food* | 2.50 | 3.00 | 0.57 | 1.51 | 2.00 | 1.87 | 2.39 | 0.30 | 0.25 | 0.50 | 1.71 | 2.98 | 2.80 | 3.90 |
| NPI- symptoms total score | 24.75 | 18.01 | 17.14 | 17.61 | 15.80 | 16.56 | 1.91 | 0.38 | 16.25 | 9.78 | 10.14 | 5.58 | 31.20 | 22.87 |
| NPI-distress total score | 6.25 | 7.59 | 8.57 | 10.20 | 5.80 | 4.82 | 0.11 | 0.95 | 6.25 | 6.24 | 3.14 | 6.23 | 13.60 | 9.50 |
| QoL-AD (caregivers) | 31.75 | 4.35 | 28.71 | 6.60 | 26.60 | 5.46 | 2.12 | 0.35 | 30.50 | 5.07 | 30.14 | 8.82 | 24.60 | 5.73 |
| QoL-AD (PwD) | 38.50 | 1.91 | 35.14 | 4.98 | 37.40 | 3.36 | 1.77 | 0.41 | 41.00 | 1.82 | 35.86 | 2.97 | 32.80 | 5.76 |
| CSDD | 5.25 | 2.06 | 7.29 | 5.99 | 5.40 | 4.16 | 0.11 | 0.95 | 4.25 | 1.71 | 4.43 | 2.51 | 6.60 | 3.78 |

Notes. ADAS-Cog: Alzheimer’s Disease Assessment Scale -Cognitive subscale; MOCA: Montreal Cognitive Assessment; NPI: Neuropsychiatric Inventory (scores on each symptom refers to mean frequency x severity); QoL-AD: Quality of Life - Alzheimer's Disease scale; CSDD: Cornell scale for depression in dementia.

Table S3. Descriptive statistics for the gain scores [(post-test – pre-test)] for each measure of interest by treatment groups (TG1: structured horticultural activities; TG2: structured horticultural activities + cognitive stimulation), and results from the Mann-Whitney test for the differences between the two treatment groups on the gain scores for each measure of interest, for the total sample and for participants with mild-to-moderate dementia only.

|  | Total  sample | | | | | | Mild-to-moderate  dementia only | | | | | |
| --- | --- | --- | --- | --- | --- | --- | --- | --- | --- | --- | --- | --- |
|  | TG1 | | TG2 | | Gain scores differences | | TG1 | | TG2 | | Gain scores differences | |
|  | M | SD | M | SD | Z | *p* | M | SD | M | SD | Z | *p* |
| ADAS-Cog |  |  |  |  |  |  | -4.28 | 13.08 | -3.00 | 2.41 | 0.00 | 1.00 |
| MoCA |  |  |  |  |  |  | 1.00 | 3.92 | 1.43 | 1.40 | -0.58 | 0.56 |
| *NPI - Delirium* | -1.71 | 4.54 | -1.12 | 1.73 | -0.65 | 0.51 | -3.00 | 6.00 | -1.29 | 1.80 | -0.20 | 0.84 |
| *NPI - Hallucinations* | 0.00 | 0.00 | -0.25 | 2.71 | 0.00 | 1.00 | 0.00 | 0.00 | -0.29 | 2.93 | 0.00 | 1.00 |
| *NPI - Agitation* | 0.14 | 4.38 | -2.25 | 3.58 | -1.11 | 0.27 | 2.75 | 2.36 | -2.71 | 3.59 | -2.28 | 0.02 |
| *NPI - Disphoria* | 1.29 | 4.11 | -1.50 | 2.93 | -0.86 | 0.39 | 0.25 | 4.03 | -1.43 | 3.16 | -0.10 | 0.92 |
| *NPI - Anxiety* | -2.14 | 4.74 | -0.62 | 2.33 | -0.31 | 0.76 | -3.75 | 5.68 | -0.71 | 2.50 | -1.03 | 0.30 |
| *NPI - Euphoria* | 0.57 | 1.51 | -0.13 | 0.35 | -1.37 | 0.17 | 1.00 | 2.00 | -0.14 | 0.38 | -1.40 | 0.16 |
| *NPI - Apathy* | -2.14 | 4.10 | 0.12 | 3.80 | -1.10 | 0.27 | -1.25 | 1.89 | 0.14 | 4.10 | -0.72 | 0.47 |
| *NPI - Dishinibition* | -0.29 | 0.95 | -0.62 | 0.92 | -0.72 | 0.47 | -0.75 | 0.96 | -0.71 | 0.95 | -0.10 | 0.92 |
| *NPI - Irritability* | 1.00 | 3.42 | -0.75 | 0.89 | -1.21 | 0.23 | 2.25 | 4.19 | -0.57 | 0.79 | -1.46 | 0.14 |
| *NPI - Motion* | -3.57 | 5.16 | -0.75 | 1.75 | -1.53 | 0.13 | -3.75 | 6.85 | -0.86 | 1.86 | -1.09 | 0.27 |
| *NPI - Sleep* | 0.57 | 1.51 | 0.38 | 0.92 | -0.28 | 0.78 | 0.00 | 0.00 | 0.43 | 0.98 | -0.88 | 0.38 |
| *NPI - Food* | -2.00 | 2.65 | 1.00 | 3.42 | -1.74 | 0.08 | -2.25 | 3.30 | 1.14 | 3.67 | -1.26 | 0.21 |
| NPI- symptoms total score | -8.29 | 14.17 | -6.50 | 17.08 | -0.23 | 0.82 | -8.50 | 19.98 | -7.00 | 18.39 | -0.19 | 0.85 |
| NPI-distress total score | -0.57 | 6.24 | -4.75 | 8.43 | -0.76 | 0.45 | 0.00 | 8.37 | -5.43 | 8.87 | -0.66 | 0.51 |
| QoL-AD (caregivers) | -1.29 | 2.69 | 2.00 | 6.97 | -1.58 | 0.12 | -1.25 | 1.89 | 1.43 | 7.32 | -1.15 | 0.25 |
| QoL-AD (PwD) |  |  |  |  |  |  | 2.50 | 1.73 | 0.71 | 3.64 | -0.65 | 0.51 |
| CSDD | -2.43 | 3.10 | -2.62 | 4.24 | -0.23 | 0.81 | -1.00 | 2.16 | -2.86 | 4.53 | -0.48 | 0.63 |

Notes. ADAS-Cog: Alzheimer’s Disease Assessment Scale -Cognitive subscale; MOCA: Montreal Cognitive Assessment; NPI: Neuropsychiatric Inventory (scores on each symptom refers to mean frequency x severity); QoL-AD: Quality of Life - Alzheimer's Disease scale; CSDD: Cornell scale for depression in dementia.

Table S4. Descriptive statistics for the behavioral symptoms, quality of life and depression measures by assessment session (pre-test, post-test) and group (TG: structured horticultural activities; CG: treatment-as-usual), and results from the Mann-Whitney test for the differences between groups at pre-test.

|  | Pre-test | | | |  | | Post-test | | | |
| --- | --- | --- | --- | --- | --- | --- | --- | --- | --- | --- |
|  | TG | | CG | | Differences at baseline | | TG | | CG | |
|  | M | SD | M | SD | Z | p | M | SD | M | SD |
| *NPI - Delirium* | 1.87 | 3.60 | 0.11 | 0.33 | -1.33 | 0.18 | 0.47 | 1.55 | 0.89 | 2.02 |
| *NPI - Hallucinations* | 0.67 | 1.80 | 0.00 | 0.00 | -1.12 | 0.26 | 0.53 | 2.07 | 2.00 | 4.24 |
| *NPI - Agitation* | 2.87 | 3.29 | 1.89 | 2.36 | -0.61 | 0.54 | 1.73 | 1.91 | 5.22 | 5.28 |
| *NPI - Disphoria* | 1.20 | 2.21 | 0.22 | 0.44 | -0.93 | 0.35 | 1.00 | 2.48 | 1.78 | 4.05 |
| *NPI - Anxiety* | 1.87 | 3.29 | 0.89 | 1.53 | -0.63 | 0.53 | 0.53 | 0.99 | 1.11 | 1.76 |
| *NPI - Euphoria* | 0.07 | 0.26 | 0.00 | 0.00 | -0.78 | 0.44 | 0.27 | 1.03 | 0.78 | 1.20 |
| *NPI - Apathy* | 3.13 | 4.16 | 2.56 | 4.39 | -0.20 | 0.85 | 2.20 | 3.59 | 2.56 | 3.97 |
| *NPI - Dishinibition* | 0.87 | 1.19 | 1.22 | 2.22 | -0.07 | 0.95 | 0.40 | 1.12 | 1.00 | 2.00 |
| *NPI - Irritability* | 1.20 | 1.78 | 1.11 | 2.02 | -0.35 | 0.73 | 1.27 | 2.22 | 2.44 | 3.97 |
| *NPI - Motion* | 4.67 | 5.18 | 4.44 | 5.81 | -0.32 | 0.75 | 2.60 | 3.78 | 3.33 | 4.24 |
| *NPI - Sleep* | 0.33 | 0.72 | 0.67 | 2.00 | -0.41 | 0.68 | 0.80 | 1.70 | 4.44 | 5.81 |
| *NPI - Food* | 1.33 | 2.09 | 1.78 | 2.27 | -0.45 | 0.66 | 0.93 | 2.12 | 2.00 | 3.16 |
| NPI- symptoms | 20.07 | 15.38 | 14.89 | 11.95 | -1.11 | 0.27 | 12.73 | 7.42 | 27.56 | 18.35 |
| NPI-distress | 6.53 | 8.25 | 4.89 | 3.72 | -0.39 | 0.70 | 3.73 | 5.42 | 9.56 | 8.58 |
| QoL-AD (cgv) | 29.33 | 5.50 | 26.00 | 4.63 | -1.50 | 0.13 | 29.80 | 7.28 | 24.67 | 4.44 |
| CSDD | 6.67 | 4.58 | 5.22 | 3.93 | -0.75 | 0.45 | 4.13 | 1.96 | 6.22 | 4.43 |

Notes. ADAS-Cog: Alzheimer’s Disease Assessment Scale -Cognitive subscale; MOCA: Montreal Cognitive Assessment; NPI: Neuropsychiatric Inventory (scores on each symptom refers to mean frequency x severity); QoL-AD: Quality of Life - Alzheimer's Disease scale; CGV: caregivers; CSDD: Cornell scale for depression in dementia.

Table S5. Descriptive statistics for the measure of interest by assessment session (pre-test, post-test) and group (TG: structured horticultural activities; CG: treatment-as-usual), and results from the Mann-Whitney test for the differences between groups at pre-test.

|  | Pre-test | | | |  | | Post-test | | | |
| --- | --- | --- | --- | --- | --- | --- | --- | --- | --- | --- |
|  | TG | | CG | | Differences at baseline | | TG | | CG | |
|  | M | SD | M | SD | Z | p | M | SD | M | SD |
| ADAS-Cog | 50.53 | 7.20 | 44.66 | 10.96 | -0.98 | 0.33 | 47.02 | 7.57 | 43.62 | 10.72 |
| MoCA | 14.17 | 2.91 | 16.36 | 2.82 | -1.36 | 0.17 | 15.44 | 3.69 | 13.96 | 4.26 |
| *NPI - Delirium* | 2.55 | 4.03 | 0.20 | 0.45 | -1.17 | 0.24 | 0.64 | 1.80 | 1.20 | 2.68 |
| *NPI - Hallucinations* | 0.91 | 2.07 | 0.00 | 0.00 | -0.99 | 0.33 | 0.73 | 2.41 | 3.60 | 5.37 |
| *NPI - Agitation* | 2.45 | 3.24 | 0.80 | 1.79 | -0.98 | 0.33 | 1.73 | 1.95 | 3.60 | 5.37 |
| *NPI - Disphoria* | 1.45 | 2.51 | 0.40 | 0.55 | -0.39 | 0.70 | 0.64 | 1.80 | 3.20 | 5.22 |
| *NPI - Anxiety* | 2.09 | 3.78 | 1.60 | 1.82 | -0.37 | 0.72 | 0.27 | 0.65 | 1.20 | 1.79 |
| *NPI - Euphoria* | 0.09 | 0.30 | 0.00 | 0.00 | -0.67 | 0.50 | 0.36 | 1.21 | 0.40 | 0.89 |
| *NPI - Apathy* | 2.82 | 4.17 | 4.60 | 5.18 | -1.07 | 0.29 | 2.45 | 4.08 | 1.60 | 2.19 |
| *NPI - Dishinibition* | 0.91 | 1.14 | 1.40 | 2.61 | -0.13 | 0.90 | 0.18 | 0.60 | 1.20 | 2.68 |
| *NPI - Irritability* | 0.91 | 1.30 | 0.40 | 0.89 | -0.76 | 0.45 | 1.36 | 2.38 | 4.00 | 4.90 |
| *NPI - Motion* | 4.18 | 4.71 | 3.20 | 5.22 | -0.59 | 0.55 | 2.27 | 3.80 | 2.80 | 3.35 |
| *NPI - Sleep* | 0.27 | 0.65 | 1.20 | 2.68 | -0.25 | 0.80 | 0.55 | 1.04 | 5.60 | 6.07 |
| *NPI - Food* | 1.27 | 2.24 | 2.00 | 1.87 | -0.72 | 0.47 | 1.18 | 2.44 | 2.80 | 3.90 |
| NPI- symptoms total score | 19.91 | 17.27 | 15.80 | 16.56 | -0.85 | 0.40 | 12.36 | 7.54 | 31.20 | 22.87 |
| NPI-distress total score | 7.73 | 9.00 | 5.80 | 4.82 | 0.00 | 1.00 | 4.27 | 6.12 | 13.60 | 9.50 |
| QoL-AD (caregivers) | 29.82 | 5.85 | 26.60 | 5.46 | -1.14 | 0.26 | 30.27 | 7.38 | 24.60 | 5.73 |
| QoL-AD (PwD) | 36.36 | 4.34 | 37.40 | 3.36 | -0.46 | 0.65 | 37.73 | 3.61 | 32.80 | 5.76 |
| CSDD | 6.55 | 4.89 | 5.40 | 4.16 | -0.11 | 0.91 | 4.36 | 2.16 | 6.60 | 3.78 |

Notes. ADAS-Cog: Alzheimer’s Disease Assessment Scale -Cognitive subscale; MOCA: Montreal Cognitive Assessment; NPI: Neuropsychiatric Inventory (scores on each symptom refers to mean frequency x severity); QoL-AD: Quality of Life - Alzheimer's Disease scale; CGV: caregivers; PwD: people with dementia; CSDD: Cornell scale for depression in dementia.
